# Recent advances on the mechanism of acupuncture in the treatment of subjective tinnitus

**DOI:** 10.3389/fnsys.2025.1523761

**Published:** 2025-06-02

**Authors:** Feifei Shi, Ziyu Ye, Bixiang Zha, Weixian Wu, Yating Zhang, Leiyu Yu, Wanting Liu, Yan Rong, Jun Yang

**Affiliations:** ^1^Medicine Gastroenterology Department, Second Affiliated Hospital of Anhui University of Traditional Chinese Medicine, Hefei, China; ^2^The First Clinical Medical Collage of Anhui University of Chinese Medicine, Hefei, China; ^3^Department of Acupuncture and Moxibustion, First Affiliated Hospital of Anhui University of Traditional Chinese Medicine, Hefei, China

**Keywords:** acupuncture, subjective tinnitus, microcirculation of the inner ear, autoimmune inflammation, nerve cells, brain network regulation

## Abstract

**Objective:**

To review and organize the research results on the mechanism of action of acupuncture in the treatment of subjective tinnitus over the past 30 years. This will provide a reference basis for the clinical acupuncture treatment of subjective tinnitus.

**Methods:**

Computer searches of PubMed, China National Knowledge Infrastructure (CNKI), and China Science and Technology Journal Database (CCD) were conducted to collect and organize literature on the research on the mechanism of action of acupuncture in the treatment of subjective tinnitus. The searches were limited to the period from January 1, 1995 to July 31, 2024. The literature was then summarized and analyzed in terms of the blood circulation of the inner ear, immuno-inflammation, and neural cells to elaborate on the potential mechanism of action of acupuncture. The objective of this study was to describe the potential mechanism of action of acupuncture. The final results yielded 36 research papers related to subjective tinnitus and the mechanism of action of acupuncture. The identified mechanisms are as follows: the enhancement of local microcirculation in the inner ear by regulating the blood supply of the vertebrobasilar artery may improve the inner ear’s blood supply obstacle. Additionally, the reduction of immuno-inflammatory factors in the inner ear may reduce the toxicity of the cochlea’s hair cells and protect them. The modulation of 5-hydroxytryptamine receptors in the cochlear nucleus may serve to protect spiral ganglion neurons from damage. Additionally, the modulation of the thalamus and the functional reorganization of the auditory cortex and synaptic network may contribute to the achievement of therapeutic effects.

**Conclusion:**

While acupuncture has demonstrated clinical efficacy in the treatment of subjective tinnitus, the underlying mechanism of action remains poorly understood. In the future, there is a need to accelerate the application of modern advanced technology and multidisciplinary cross-fertilization, as well as to conduct in-depth and systematic investigations into the mechanisms of acupuncture effects. This will provide an objective basis for clinical treatment.

## 1 Introduction

Subjective tinnitus constitutes an anomalous auditory perception in the absence of external acoustic sources within the cranium. One of the challenging clinical conditions commonly encountered in otolaryngology practice. The overall prevalence of subjective tinnitus in adults is approximately 14.4%, potentially affecting over 740 million adults worldwide (Jarach et al., 2022). Persistent and bothersome tinnitus exerts mental, tinnitus exerts mental, psychological, and behavioral impacts on patients, leading to diverse manifestations of negative psychological states. This adverse mental condition and tinnitus often create a detrimental feedback loop, potentially escalating to severe cases with suicidal inclinations (Clifford et al., 2020). Tinnitus, conceptualized as a hallucinatory perception, exhibits strong subjective features with an etiology that remains elusive; Consequently, its underlying mechanisms are ambiguous. Presently, clinical interventions for tinnitus predominantly encompass pharmacological treatment, cognitive-behavioral therapy, and phoniatric treatment among others. However, the 2019 European Multidisciplinary Guidelines for Tinnitus Management discourage pharmacological interventions and emphasize the primacy of cognitive-behavioral therapy, which modifies patients’ behavioral reactions and their cognitions related to the tinnitus or its symptoms. Acupuncture, an ancient therapeutic modality of the Chinese nation, has been extensively practiced by medical professionals across various dynasties. As quoted in “The Spirit Pivot The Thorn Section, True Evil,” “The blind man cannot see, the deaf man cannot hear. He who experiences this prick shall find himself in the sun, in the palace of hearing, in his eyes.” Studies have demonstrated the efficacy of acupuncture in the management of subjective tinnitus, resulting in favorable clinical outcomes (Cai et al., 2024). Systematic reviews and meta-analyses of randomized controlled trials indicate that acupuncture can diminish and even eliminate the intensity of tinnitus, alleviate tinnitus-related insomnia, and enhance the quality of life for sufferers (Zha V. B. et al., 2024). However, the pathophysiological mechanism of tinnitus is still unclear, and the mainstream view is that the core of its pathogenesis is related to peripheral auditory damage that triggers compensatory remodeling of the center and maladaptive neuroplasticity (Kang and Zheng, 2024). At the cellular level, a decrease in cochlear afferent nerve input (e.g., hair cell degeneration or auditory neuropathy) induces an increase in spontaneous firing rate and synchronization of neurons in the dorsal cochlear nucleus (DCN) (Shore et al., 2016; Teismann et al., 2014), and a concomitant decrease in inhibitory neurotransmitter (e.g., GABA)-driven and excitatory imbalance leads to neuronal hyper-excitability (Richardson et al., 2012), which disrupts the excitatory-inhibitory homeostasis and creates a neurological basis for tinnitus (Moller, 2016; Bauer, 2018; Kalappa et al., 2014). At the systemic level, cochlear deafferentation triggers topographic reorganization and gain enhancement in the auditory cortex (AC) through activation of the DCN and aberrant activity in the inferior colliculus (IC), which integrates auditory and limbic system signals via the medial geniculate body (MGB) (Henton and Tzounopoulos, 2021; Xia et al., 2020; Eggermont, 2015), whereas aberrant functional connectivity in non-auditory areas such as the anterior cingulate cortex (ACC), insula, and prefrontal lobes further mediate the emotional and attention dysregulation (Vanneste and De Ridder, 2012) and the mechanisms by which acupuncture alleviates tinnitus are not well-documented. Consequently, acupuncture is not recommended by the European Multidisciplinary Tinnitus Guidelines. Hence, investigating the mechanisms of acupuncture in the treatment of subjective tinnitus holds significant potential.

## 2 Acupuncture promotes microcirculation in the inner ear region

The blood supply to the ear primarily originates from two branches of the external carotid artery: the maxillary artery and the superficial temporal artery, which together provide nourishment to the outer ear and parts of the middle ear (Nguyen and Duong, 2024). The inner ear, an organ of remarkable complexity, is responsible for the encoding of sound, movement, and spatial orientation. The integrity of its blood supply is fundamental to the growth, maturation, and viability of the inner ear’s tissue and its physiological functions. The labyrinthine artery, the sole terminal blood vessel providing circulation to the inner ear, branches into three main components: the anterior vestibular artery, the cochlear artery, and the vestibulo-cochlear artery. This artery traverses the internal auditory canal, accompanying the cochlear vestibular nerve and emerges from either the anterior inferior cerebellar artery’s outer loop (predominantly, approximately 90%) or the basilar artery (around 10%). It measures with an average diameter of 0.2 ± 0.05 mm (Matsubara, 2024), demonstrating its delicate nature. Consequently, conditions such as vasospasms, impairment of blood flow, increased blood viscosity, and the formation of microthromboses can disrupt the circulation of the labyrinthine artery, resulting in inner ear ischemia, hypoxia, neural injury, and the onset of tinnitus symptoms. Research indicates that occlusive disorders within the vertebrobasilar artery system can adversely affect the regulatory mechanisms for inner ear blood flow, manifesting as symptoms of dizziness, tinnitus, and hearing loss (Ueda and Matsunaga, 1995). The interconnection between these systems may be mediated through the vascularity supply to trigeminal sensory neurons (Vass et al., 2001). The effects of acupuncture on microcirculation and hemorheology have long been the focus of research on the mechanism of acupuncture, such as the regulation of vasoactive substances, neurotransmitters, signaling pathways and oxidative stress (Zhang et al., 2024). Acupuncture can promote the improvement of hemodynamics, the release of vasoactive substances, the formation of new blood capillary refill time, accelerate the speed of cerebral blood flow in the basilar artery and mesencephalic artery, and effectively improve cerebral microcirculation (Ke et al., 2021). This may be related to the regulation of serum levels of serotonin, vascular endothelial growth factor and calcitonin gene-related neurohumoral regulation and enhance the local microcirculation of the inner ear to improve the blood supply disorder of the inner ear (Zheng et al., 2006). Cai et al. (2019) measured bilateral auricular temperatures in tinnitus patients before and after acupuncture using infrared thermography. The results showed a significant reduction in temperature differences between the bilateral ears post-acupuncture, suggesting that acupuncture may enhance cochlear microcirculatory stability. In addition, abnormal lipid levels have also become a new biomarker of subjective tinnitus. Acupuncture treatment can not only address hyperlipidemia, but also reduce the severity of tinnitus (Ali, 2023).

## 3 Acupuncture modulates the immunological and inflammatory responses in the inner ear

The inner ear engages in a complex interplay with the systemic immune system, primarily via lymphatic drainage and vascular circulation, to protect itself against infections and stressors, including auditory trauma. Allergens, pathogens, and autoimmune responses can precipitate middle ear effusions, eustachian tube mucosal inflammation, or ossicular chain (the auditory ossicular chain consists of the following three small bones - the malleus, incus, and stapes) involvement, disrupting the inner ear’s homeostasis and precipitating sensorineural hearing loss and tinnitus. A constellation of immune-inflammatory mechanisms, potentially involving genetic variants, nuclear factor-κB-driven inflammation, interleukin-1β, tumor necrosis factor-*α*, and other cytokines, may partially elucidate the pathogenesis of acute tinnitus. A comprehensive literature review has revealed no published studies investigating the correlation between immune responses and chronic tinnitus.” I think you should also say it in the text. In addition to neurophysiological alterations within the central nervous system, it may be intertwined with chronic stress (Becker et al., 2022). secondary to the priming of the peripheral inflammatory response (Basso et al., 2022). Investigations into the modulatory impact of acupuncture on the immune system have corroborated its immunomodulatory effects on infectious and allergic conditions. Research has delved into the mechanisms that underpin the amelioration of inflammation, which include vagus nerve activation, modulation of the toll-like receptor 4/NF-κB signaling cascade, macrophage polarization, activation of the mitogen-activated protein kinase pathway, and engagement of the cholinergic anti-inflammatory pathway. According to the study conducted by Xiaofeng (2018), serum IL-6 and TNF-*α* levels in the acupuncture group showed significant reductions post-treatment compared to pre-treatment levels. Furthermore, the extent of these reductions was positively correlated with the decrease in the total score of the Hamilton Depression Rating Scale. These findings suggest that the regulation of inflammatory cytokines through electroacupuncture may constitute one of the mechanisms underlying the treatment of depressive symptoms associated with occupational noise-induced hearing loss. Acupuncture has oxygen therapy to manage patients with autoinflammatory vasculitis and bilateral sensorineural hearing loss, resulting in a 20 dB improvement in hearing thresholds after a month been shown to augment the population of cochlear hair cells, mitigate oxidative stress in mice, and alleviate the effects of transgenic ototoxicity via the nuclear factor erythroid 2-related factor 2 signaling pathway (Yu et al., 2022). Furthermore, it can counteract ototoxicity Induced by Gentamicin in Mice by upregulating the expression of neurotrophic factor 3 in the nucleus of the inferior colliculus, thereby shielding cochlear hair cells (Zhou et al., 2017). Electroacupuncture has been reported to enhance the expression of aquaporin 1 in the cochlea, which in turn increases the concentrations of potassium and calcium ions, thereby attenuating endolymphatic edema in guinea pigs (Jiang et al., 2015). Moreover, acupuncture has been observed to alter liver lipid metabolism by diminishing leptin sensitivity and to modulate the immune response, thereby alleviate depression-like behaviors (Jung et al., 2021).

## 4 Acupuncture is effective in mitigating damage to the periauricular nerves

Nerve fibers, blood vessels, and lymphatic vessels are disseminated extensively within and beyond the skin of the auricle. Nerves of the ear, which are interconnected with blood vessels, extend from the basal region to the peripheral areas, traversing lymphatic vessels to establish an integrated network of nerves, blood vessels, and lymphatic vessels that function in concert. Cochlear hair cell injury with subsequent degeneration of the associated nerve fibers (Wang et al., 2024), originating from factors such aging, or other sources, can precipitate spontaneous firing patterns. These patterns may result in the central nervous system experiencing reduced inhibition and heightened activity, culminating in the generation of phantom perceptions. The primary destruction of the cochlear nerve encompasses lesions in the spiral ganglion neurons, the cochlear vestibular nerve, and various cochlear synaptic administered (Zhou et al., 2009) nerve growth factor via injections to the Yifeng and Wangu acupoints for the treatment of sound-perceiving nerve deafness and tinnitus, yielding superior outcomes compared to the treatment efficacy observed in the Western medication group, which ingested Sipyrine and adenosine triphosphate orally. Chang et al. (2021) electroacupuncture, examining the resultant reduction in spiral ganglion neuron density and the auditory status of the rats. Their findings indicated that electroacupuncture exerted a beneficial influence on mitigated spiral ganglion neuron damage and aiding in the recovery of hearing. Kang et al. (1998) electroacupuncture treatment preserves the integrity of lysosomes in cochlear hair cells, preventing Serotonin, as a crucial neurotransmitter, modulates auditory signals in the cochlear nucleus through various receptor subtypes (Dai et al., 2015) electroacupuncture at the Yifeng point could augment the expression level of 5-hydroxytryptamine receptor 1b mRNA in the cochlear nucleus of mice exposed to mobile phone radiation, while concurrently suppressing the expression of 5-hydroxytryptamine receptor 2c mRNA. The depth of acupuncture is a critical factor in modulates the autonomic nervous system balance in individuals experiencing tinnitus (Lee et al., 2023). Research indicates that autonomic dysfunction is a risk factor for subjective (Tu et al., 2019) divided 30 patients into either a deep or shallow acupuncture group, administered acupuncture to both and assessing changes in heart rate variability and Tinnitus Handicap Inventory (THI) pre- and post-treatment. The study yielded evidence suggesting that deep-prick acupuncture significantly improved heart rate variability (HRV) and that there was a physiological correlation between autonomic modulation and HRV, suggesting that acupuncture may alleviate tinnitus symptoms by modulating sympathetic-parasympathetic balance.

## 5 Acupuncture is known to modulate brain architecture and neural connectivity

Currently, scholarly inquiries into the etiology of tinnitus are predominantly concentrated on the peripheral auditory pathways and the central auditory, as well as the non-auditory systems. It is a consensus among professionals that damage to the peripheral auditory pathways often elicits a central compensatory response, which can lead to a persistent state of tinnitus. This condition is characterized by a dynamic interplay of abnormal local neural activity and dysfunctional networking within multiple brain systems. The advent of contemporary neuroimaging technologies has furnished a pivotal “visualization” tool for elucidating the mechanisms underlying the therapeutic efficacy of acupuncture in mitigation of tinnitus. Researchers such as Yu et al. (2023); Fan et al. (2024) near-infrared spectroscopy (fNIRS) to delineate the discrepancies in neural activity within the cerebral cortex during auditory stimulatory tasks, thereby differentiating subjective neural tinnitus cerebral cortex of tinnitus sufferers using fNIRS, documenting an increase in the concentration of oxyhemoglobin in the temporal lobes of these patients concomitant with the activation of auditory cortex. Additional studies (Yang et al., 2019) have instituted auricular point electrical NMDAR positive modulator can selectively amplify the endogenous activity of NMDAR in GABAergic neurons, thereby potentiating inhibitory neurotransmission, upsetting the balance between excitation and inhibition, and potentially retarding the onset of subjective tinnitus attributable to inhibitory deficits stimulation in concert with masking techniques to modulate the BDNF/TrkB/CREB signaling cascade within the auditory cortex of tinnitus-afflicted rodents, culminating in the enhancement of the pre-pulse suppression threshold in the presence of high-frequency background noise – an effect indicative of tinnitus amelioration. Functional magnetic resonance imaging (fMRI) technology has emerged as a pivotal tool for investigating the functional dynamics and dysfunctions of the human brain, owing to its non-invasive nature, real-time imaging capabilities, and high spatial and temporal resolution. A team led by the nationally renowned traditional Chinese medicine expert, Professor Yang Jun, administered acupuncture therapy to patients experiencing subjective tinnitus, employing the principles of “calming and clearing body, activating blood channel.” The team recorded the Tinnitus Handicap Inventory (THI), Tinnitus Evaluation Questionnaire (TEQ), and Visual Analogue Scale (VAS) scores both pre- and post-acupuncture intervention, demonstrating significant clinical efficacy. Utilizing fMRI, the researchers compared alterations in brain regions exhibiting varying amplitudes of ALFF 197 (Amplitude of Low-Frequency Fluctuations) “fALFF” (Fractional Amplitude of Low-Frequency Fluctuations) values, as well as the functional connectivity among the head, body, and tail of the bilateral caudate nucleus before and after acupuncture treatment for chronic subjective tinnitus. The findings revealed that acupuncture can enhance the ALFF values in of the auditory cortex and the right superior frontal gyrus of the default mode network, facilitating functional reorganization within the auditory cortex and the frontal cortex. Additionally, the study observed a reduction in the functional connectivity between the caudate nucleus, the auditory cortex, and the limbic system, which was found to be negatively correlated with the alleviation of subjective neurological tinnitus (Zhang, 2022), along with 22 age- and gender-matched normal controls (control group) (Wei et al., 2021). Utilizing the resting-state fMRI dynamic functional connectivity analysis approach, the researchers assessed the hearing and tinnitus-related scales of the chronic subjective tinnitus patients. Their findings revealed that, in contrast to healthy individuals, patients with chronic neural tinnitus exhibited heightened temporal variability in the dynamic functional connectivity between the supplementary motor area and the medial superior frontal gyrus, which was positively correlated with hearing loss. Additionally, following acupuncture treatment, a significant reduction in the dynamic functional connectivity between the posterior cingulate gyrus and the angular gyrus was observed in chronic tinnitus patients, a change that correlated positively with the amelioration of tinnitus insula, and amygdala within the salient network, and comparatively analyzed the differences in brain networks between subjective tinnitus patients and healthy volunteers both pre- and post-acupuncture treatment. The study yielded results indicating that, subsequent to acupuncture, the brain networks of subjective tinnitus patients exhibited notable enhancements. Decreases in functional connectivity (FC) were observed in the right anterior cingulate gyrus, left lingual gyrus, and left thalamus. Similar reductions in FC were found in the left anterior cingulate gyrus, right middle frontal gyrus, and right. Superior frontal gyrus, as well as in the right insula and right middle frontal gyrus. Furthermore, a decrease in FC was detected in the left insula, left middle frontal gyrus, and right superior frontal gyrus, changes that positively correlated with the improvement of Zhang et al., (2022, 2024); Zha B. et al., (2024) tinnitus symptoms.

## 6 Synopsis and future perspective

The comprehensive analysis indicates that acupuncture therapy offers a wide variety of clinical acupoint selection and operational techniques for subjective tinnitus treatment. Nonetheless, the subjective nature of tinnitus presents a challenge, as there are no universally accepted objective criteria for evaluation, coupled with an enigmatic pathophysiological underpinning. Presently, the therapeutic emphasis is placed on modulating vertebral-basilar artery blood supply and enhancing inner ear circulation, aimed at diminishing immune-inflammatory mediators within the cochlea and safeguarding cochlear hair cells. Additionally, there is a focus on regulating neurotrophic support in the cochlear nucleus to mitigate damage to the spiral ganglion neurons, as well as on reorganization of the thalamic-auditory cortex/amygdala network function. To strengthen the robust evidence-base of acupuncture in the management of subjective tinnitus and elucidate the underlying mechanisms of its effectiveness, future endeavors may be directed toward the following paths: (1) conducting standardized, multi-center, randomized controlled trials evaluating the efficacy of acupuncture for subjective tinnitus; (2) exploring the therapeutic mechanism of acupuncture for tinnitus through comprehensive neuroimaging and Doppler ultrasound studies, based on clinical outcomes; (3) the modern biological mechanisms of acupuncture in tinnitus treatment was studied by means of extrinsic somatic electrophysiology and patch clamp technique from macroscopic brain imaging down to mesoscopic neuroelectrophysiology across species. Limitations of this study include the scope of literature screening and methodologic differences, and future systematic evaluations are needed to provide support for higher levels of evidence.
